# Early Intervention is Important to Prevent Sensitization to New Allergens

**DOI:** 10.3390/medsci6040114

**Published:** 2018-12-11

**Authors:** Tetsuya Terada, Ryo Kawata

**Affiliations:** Department of Otolaryngology, Osaka Medical College, 2-7 Daigakumachi Takatsuki, Osaka 569-8686, Japan; oto034@osaka-med.ac.jp

**Keywords:** allergic rhinitis, early intervention, antigen exposure, immunotherapy

## Abstract

We review current management for allergic rhinitis and possible new treatments for this condition. Management of allergic rhinitis includes promotion of protective factors, avoidance of allergens, and possibly immunotherapy. In recent years, the incidence of allergic rhinitis has increased in many countries. Early intervention at different stages is an important part of management. Allergic disease in infants has been described as the allergic march, commencing with atopic dermatitis accompanied by infantile asthma and progressing to perennial allergic rhinitis induced by house dust mite allergy. In order to prevent polysensitization, allergen-specific immunotherapy should probably be initiated at an earlier age, especially in children with rhinitis who show monosensitization to house dust mite antigens.

## 1. Introduction

Allergic rhinitis (AR) is a worldwide problem, and some studies have suggested that its incidence is increasing [1,2]. Allergic rhinitis is an important public health issue because of its wide prevalence and impact on the quality of life, school performance, and productivity at work [3].

Allergic rhinitis is the fifth most common chronic disease in the United States, affecting 10%–30% of the adult population and up to 40% of children. However, the impact of AR on health and the economy has been grossly underestimated historically, and it has only recently been recognized as a serious epidemic. In the US, the direct medical cost of AR was $6.1 billion in 2000 and it rose to $11.2 billion by 2005. Moreover, AR has an estimated annual impact on productivity of $600 per US employee, which is higher than for diabetes, coronary heart disease, and asthma. Indirect losses due to AR are also a heavy burden for developing countries. In eight countries from the Asia Pacific region, the annual cost of AR per patient, including loss of productivity, was reported to range from US$184 to US$1189. Thus, the cost burden of AR is enormous, but is generally under-appreciated [4].

There have been relatively few studies of AR in children compared with adults, so little is known about the onset and natural history of seasonal AR in school children, including its prevalence and incidence rate. In addition, it is rare for studies to follow a cohort of children over several years [5,6,7]. Evidence has been presented that initial symptoms of AR can occur during the first year of life. Atopic diseases develop as the result of complex interaction between genetic factors, environmental allergens, and adjuvant factors that include tobacco smoke, air pollution, and infections. Preventive measures for these diseases include reducing exposure to allergens and adjuvant risk factors, promotion of protective factors, and medication.

The infants with family history of allergy have a high risk of developing atopic diseases and it is thought to be the early intervention is necessary to such children particularly.

In addition, the upper and lower airways are linked from anatomical, histological, and immunological functions in one part of the airway, forming a united airway system. Therefore, it is suggested to consider the early interventions for AR in children to prevent asthma. 

This review evaluates early intervention for AR, such as breastfeeding, avoidance of allergens, and antigen-specific immunotherapy.

## 2. Incidence of Allergic Rhinitis in Children

Hill et al. [8] conducted a study of 29,662 children based on health care records and reported that the incidence of physician-diagnosed AR was 1% during the first year of life. Subsequently, the annual incidence of AR ranged from 3.6% to 4.5% between one and five years of age, and the highest incidence was found at two to three years. These findings broadly correspond with the estimated annual incidence of 3% to 4% for seasonal AR between three and seven years of age in a birth cohort of 1314 German children [9]. It was reported that AR often first occurs in childhood and its prevalence increases with age [10,11,12]. The International Study of Asthma and Allergies in Childhood (ISAAC) was a global survey on the incidence of allergic diseases in children from two age groups (six to seven years and 13 to 14 years), which revealed that rhinoconjunctivitis symptoms showed a higher prevalence in the older group. According to the second ISAAC survey (ISAAC Phase Three 1999–2004), the worldwide incidence of rhinoconjunctivitis was 8.3% in the six to seven years age group (inter-country range, 1.8% to 24.2%), while it was 15.1% (4.5% to 45.1%) in the 13 to 14 years age group [13]. According to a recent meta-analysis of all studies performed by the ISAAC protocol (involving 1,430,329 children from 0 to 18 years), the overall incidence of AR is 12.66% [14].

## 3. Challenges Related to Epidemiological Surveys of Pediatric Allergic Rhinitis

It is difficult to perform an epidemiological survey of pediatric AR. Because AR is an antigen-specific immuglobulin E (IgE) antibody-mediated type I allergic disease, confirmation of antigen-specific IgE antibody is important for diagnosis, in addition to assessment of symptoms and nasal mucosal findings. However, the reported symptoms of infants and children are inaccurate, so diagnosis is inevitably based on questionnaire surveys of their parents. Moreover, nasal mucosal findings are hard to obtain and it is difficult to perform invasive tests in children compared with adults. Finally, the decision to seek medical care is usually made by the parents, raising the possibility that many children with AR are not seen by doctors.

The incidence of AR peaks between infancy and adolescence. A study on the prevalence of specific IgE antibody revealed that sensitization to inhaled allergens did not induce symptoms in infancy, but symptoms developed with age [15].

Ozasa et al. [16,17] performed an epidemiological survey among elementary and junior high school students in a rural region of Kyoto Prefecture to clarify the prevalence of Japanese cedar-specific IgE antibody and changes in the prevalence of Japanese cedar pollinosis associated with the airborne Japanese cedar pollen count. When a RAST score of 1 or higher was defined as indicating positivity for Japanese cedar- and mite-specific IgE antibody, approximately 50% and 40% of the subjects were positive, respectively. When observation was initiated in 1994, the prevalence of anti-Japanese cedar pollen antibody was 39%, and it increased to 52.3% in 2008. In addition, a correlation was noted between the airborne Japanese cedar pollen count and the antibody titer, and the antibody positive rate increased when testing was done immediately after the pollen season in a year with a high pollen count.

## 4. Protective Factors against Allergic Rhinitis

### Breastfeeding

It is recommended that all infants should receive breastfeeding for at least three months, irrespective of a family history of atopy [18]. However, there is no definite evidence that exclusive breastfeeding for at least the first three months of life reduces the risk of allergy or asthma, so the recommendation about exclusive breastfeeding is conditional. If the mother has classic galactosemia, active untreated tuberculosis, or HIV infection, or is receiving treatment with antimetabolites, chemotherapy agents, or radioactive isotopes, breastfeeding is not suggested until the milk is clear.

Breastfeeding provides several health benefits for the mother and child and is recommended for all infants [19]. One of the important reasons to promote breastfeeding is prevention of allergic diseases [20]. Breast milk contains multiple nutrients that support growth and promote the development of immunity [21,22], but it is unclear whether breastfeeding can help to prevent allergic diseases.

In 2015, Lodge et al. [23] performed a systematic review and meta-analysis of five cohort studies [24,25,26,27,28] and 11 cross-sectional studies [29,30,31,32,33,34,35,36,37,38,39] targeting the association of breastfeeding with AR. This meta-analysis suggested that breastfeeding had a nonsignificant protective effect against development of AR (odds ratio (OR) 0.92; 95% confidence interval (CI), 0.84 to 1.01). Age stratification revealed that the risk of AR was reduced by breastfeeding in patients under five years old (OR 0.79; 95% CI, 0.63 to 0.98), but there was no benefit after five years of age (OR 1.05; 95% CI, 0.99 to 1.12). Because of the difficulty in distinguishing between AR and viral rhinitis in young children, the authors suggested that reduction of viral respiratory infections may have been misinterpreted as improvement of rhinitis.

## 5. Avoiding Exposure to Aggravating Factors

### Environmental Tobacco Smoke

It is recommended that children and pregnant women should completely avoid environmental tobacco smoke. Smoking and passive smoking are common worldwide health problems that lead to a substantial disease burden in children and adults.

It was reported that environmental exposure to tobacco smoke is associated with chronic rhinitis and sometimes with AR [40,41]. Several studies have indicated that self-reported symptoms are elicited by exposure to smoke and are correlated with the serum cotinine level [42,43,44]. Environmental tobacco smoke has immunomodulatory effects and has been suggested to influence sensitization to allergens [45]. Many studies have linked environmental exposure to tobacco smoke with respiratory disease and allergies in children, but the impact on sensitization mediated by IgE remains less clear [46]. Some authors have reported that environmental tobacco smoke increases the risk of sensitization to any allergen [47,48], whereas others have only found an increase in the risk of sensitization to food allergens [49,50].

In addition, an inverse association [3,9,10] and null association [4] have been reported for inhalant allergens, and data about the influence of heredity are conflicting [1,3,6]. Thacher et al. [51] performed a 16-year population-based birth cohort study of 3316 children using repeated parental questionnaires. They reported that exposure to tobacco smoke during infancy without prior in utero exposure was associated with an elevated risk of sensitization to food allergens at the age of 4 years (OR 1.47, 95% CI 1.08–2.00), with a comparable risk persisting at the ages of 8 and 16 years. Longitudinal analysis indicated an overall association between environmental exposure to tobacco smoke in infancy and sensitization to food allergens up to the age of 16 years (OR 1.24, 95% CI 0.98–1.56), while maternal smoking during pregnancy was not related to food allergen sensitization up to age 16. When sensitization was associated with symptoms of allergic disease, environmental exposure to tobacco smoke during infancy was associated with an elevated risk of both eczema and food allergen sensitization (OR 1.62, 95% CI 1.20–2.18).

## 6. House Dust Mites

Various interventions are recommended to reduce early exposure to house dust mites for infants and preschool children. However, this recommendation puts relatively little emphasis on the burden and cost of employing multiple preventive measures (encasing mattresses, washing bedding and soft toys in hot water with a temperature above 55 °C, use of acaricide, smooth flooring without carpets, etc.), compared with a relatively high emphasis on a possible small reduction in the risk of developing wheezing or asthma. Thus, an alternative approach is equally reasonable for some children who have a lower risk of developing asthma and in appropriate circumstances.

Only six studies have investigated the relation between early exposure to house dust mites and AR, with most of them failing to demonstrate any association between early mite exposure and development of AR [9,52,53,54,55]. Marinho et al. [56] reported that early exposure to house dust mites was not protective against AR, while Kim et al. [57] identified exposure to spider mites as a risk factor for development of AR.

## 7. Pets

Because the timing of exposure to pet allergens early in life may have an important influence on maturation of the immune system, self-reported perinatal and newborn exposure to pets have been frequently investigated. According to a systematic review of epidemiologic studies on allergy and asthma, only 10 of the 96 studies reported avoidance of pets [58]. However, these studies often fail to account for potential confounders, such as a family history of pet allergy, which may result in pet avoidance by children who are likely to have atopy.

According to a meta-analysis of 32 studies, the prevalence of AR was lower among subjects with furry pets from cross-sectional studies, and asthma was less frequent among subjects with exposure to cats [59]. On the other hand, an extensive systematic review of 62 studies revealed differences of the associations that were dependent on the study design. In most birth cohort studies, exposure to dogs in early childhood had a protective effect against sensitization to aeroallergens, but cross-sectional studies found inconsistent associations between exposure to cats or dogs and sensitization or development of atopic diseases later in life. The impact of pet avoidance on AR is best evaluated by performing longitudinal birth cohort studies. When a systematic review of nine studies conducted in urban environments was performed to evaluate the impact of perinatal exposure to pets [60], exposure to dogs or cats/dogs was protective against allergic disease in six studies, while two studies showed an increased risk of allergy that was confined to highly atopic families. On the other hand, a cohort study of 620 children with a family history of allergic disease suggested that exposure to cats or dogs only has a protective effect for children with non-allergic fathers [61].

Thus, there is no evidence that avoiding pets in infancy prevents development of AR or sensitization to aeroallergens later in life, while early exposure may induce immune tolerance and actually reduce the risk of developing allergic disease.

## 8. Antigen Exposure Immediately after Birth

Exposure to an inhaled antigen immediately after birth, mainly within the first six months of life, promotes sensitization. According to a survey performed from 1995 to 1998 by Ozasa et al. [17], significantly more children became strongly positive when their initial Japanese cedar pollen season (February–April) was within six months of birth (i.e., infants born in fall or winter) or when they were exposed to a high airborne pollen count during the initial Japanese cedar pollen season. These findings suggest that antigen exposure within 6 months after birth, while the immune function of the infant is underdeveloped and depends on maternal immunity, may be associated with antigen sensitization.

## 9. Immunotherapy

Allergen immunotherapy (AIT) was first performed over 100 years ago in 1911 [62]. In 1998, the World Health Organization [63] and the European Academy of Allergology and Clinical Immunology (EAACI) [64] confirmed the clinical effectiveness of AIT for management of AR and asthma when standardized extracts were administered at adequate doses. According to a meta-analysis of 54 clinical trials on the efficacy of AIT for asthma, it significantly suppressed asthma symptoms, use of medications, and episodes of exacerbation. [65] Allergen immunotherapy also reduces specific bronchial hyperreactivity and may prevent asthma in patients with AR [66,67,68,69,70,71,72,73,74]. Whether AIT can prevent sensitization to other airborne allergens in patients with monosensitization was also investigated [75,76,77], and a prospective controlled study recently showed that new sensitization was suppressed for 12 years after discontinuation of preseason immunotherapy for grass pollen allergy [78].

Inai et al. [79] performed a parallel group open study in patients with AR and/or asthma who showed monosensitization to house dust mites, including 85 patients who underwent AIT and 62 who only received medication.

They found that AIT could potentially prevent new sensitization in children with rhinitis and/or asthma who displayed monosensitization to house dust mites, and they suggested that AIT should be started at an earlier age, especially to prevent polysensitization in children with rhinitis and monosensitization to house dust mites. After five years, 64/85 children (75.3%) in the AIT group did not show new sensitization versus 29/62 children (46.7%) in the control group (*p* = 0.002). In the AIT group, 15/21 patients developed sensitization to at least 1 new allergen, but only 1 of 6 patients developed sensitization to two or more new allergens. In contrast, sensitization to one new allergen occurred in 22/33 patients from the control group, and sensitization to two or more new allergens was seen in 11 patients.

Although there is no clear explanation for the lower risk of new sensitization in children receiving AIT, it has been reported that AIT affects the balance between T_H_1 and T_H_2 cells [80]. AIT also decreases production of interleukin (IL)-4 and IL-5 [81,82], increases interferon- production [83], and decreases inflammatory cells in the nose [84]. Induction of peripheral T cell tolerance by AIT is essential for it to be effective, and is initiated through increased production of IL-10 and transforming growth factor- by antigen-specific regulatory T cells. Tolerance of allergens and development of specific energy by peripheral T cells in response to IL-10 are important immune changes associated with AIT [85] that may alter or delay the natural progression of respiratory allergic diseases.

Although further investigation is required to clarify the mechanisms, we suggest that AIT is the most effective early intervention methods, and it is recommended that AIT should begin at earlier ages to prevent sensitization to new allergens.

## 10. Prior Sensitization to Mites

There is a concept regarding the development of pediatric allergic disease termed allergic march, which refers to progression of allergic diseases from atopic dermatitis in infancy to bronchial asthma and AR, although there are multiple patterns of development. The prognosis of infant-onset atopic dermatitis is generally favorable and more than 90% of children show remission or healing within several years. On the other hand, 30%–40% of these patients develop bronchial asthma in infancy, and the involvement of inhaled antigens (mainly mite antigens) leads to perennial AR at school age. In addition, many of them develop Japanese cedar pollinosis in junior high school or later.

Although spontaneous resolution of food antigen-induced atopic dermatitis can often be expected, the spontaneous resolution rate of AR is low and the importance of preventing sensitization to Japanese cedar pollen has been noted.

The titer of Japanese cedar pollen-specific IgE antibody increases after the pollen season in patients with Japanese cedar pollinosis, but the total IgE level does not change [86]. In an epidemiological survey performed by Ozasa et al. [17], an increase of total IgE with age was not noted in children sensitized to Japanese cedar pollen alone, but it was seen in children positive for both mite-specific and Japanese cedar pollen-specific IgE antibodies. The majority of antigens responsible for pediatric allergies are derived from mites and total antigen exposure is high because it occurs throughout the year. Mite antigens are also responsible for bronchial asthma. The strong association between elevation of the total IgE level and sensitization to mites may have been found because the study includes children with atopic diathesis and children with asthma. Ozasa et al. also performed a follow-up survey of antibody levels in school children, which clarified that mite-specific IgE antibody positivity was a risk factor for sensitization to Japanese cedar pollen at school age.

## 11. Pharmacologic Treatment

For the infants with nasal allergy and/or family history of allergy, it was recommended that clinicians do not administer oral H1-antihistamines for the prevention of new sensitization. This recommendation depends on a relatively high value on avoiding side effects of oral H1-antihistamines for the infants and lower value on the uncertain reduction in the risk of developing new sensitizations [18].

## 12. Conclusions

The age at which nasal allergy develops has decreased, but children are not small adults. Since pediatric nasal allergy occurs during the developmental period, including development of immune function, it cannot be considered to be the same disease as nasal allergy in adults. Once sensitization to a specific antigen has been established, nasal allergy worsens over time and spontaneous resolution cannot be expected. Thus, preventing sensitization to house dust mites is important, particularly during the preschool period, and early intervention is required to effectively manage allergic rhinitis.
